# Effect of Childhood Trauma on Emotional Distress: A Chain‐Mediated Effects of Alexithymia and Psychological Flexibility

**DOI:** 10.1002/brb3.70825

**Published:** 2025-09-07

**Authors:** Ning Wang, Jingyi Chu, Guo Chen, Xingyue Pan, Ziyue Wang, Wenjuan Wang

**Affiliations:** ^1^ School of Mental Health Bengbu Medical University Bengbu Anhui China

**Keywords:** alexithymia, anxiety, childhood trauma, depression, psychological flexibility, stress

## Abstract

**Objective:**

The study aims to explore how emotional distress is affected by childhood trauma through pathways involving alexithymia and psychological flexibility, and to construct a complete model established on this foundation, which will be provided as a theoretical theory for interventions in college students' mental health.

**Methods:**

Note that 1002 college students were assessed using Childhood Trauma Questionnaire (CTQ), The Depression Anxiety Stress Scale (DASS‐21), the 20‐item Toronto alexithymia scale (TAS‐20), and Acceptance and Action Questionnaire‐2nd Edition (AAQ‐II). After removing some non‐compliant questionnaires, the remaining 885 were used for data analysis.

**Results:**

Scores on childhood trauma, alexithymia, and psychological flexibility exhibited notable correlation with depression, anxiety, and stress; childhood trauma had chain mediation effects on depression, anxiety, and stress through alexithymia and psychological flexibility.

**Conclusion:**

Among college students, childhood trauma is positively associated with depression, anxiety, and stress, and impacts the relationship between them through the single mediation and chain mediation of alexithymia and psychological flexibility.

## Graphical abstract

The graphical abstract illustrates the associations among childhood trauma, alexithymia, psychological flexibility, and emotional distress. Childhood trauma can directly lead to emotional distress or indirectly cause it through alexithymia or psychological flexibility.

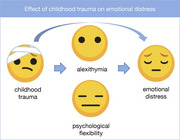

## 1 Introduction

Emotional distress refers to the negative emotions that arise in response to stress, such as depression and anxiety (Zeng et al. 2024). Depression, anxiety, and stress are three emotions that frequently coexist (Brochier and Olie 1993; Juruena et al. 2020). Clark and Watson proposed a tripartite model based on psychometric data, which comprises general distress, physiological hyperarousal (specific to anxiety), and anhedonia (specific to depression) (Clark and Watson 1991). Based on this, Lovibond established the DASS scale, which further emphasizes that depression, anxiety, and stress are correlated yet separable factors (Lovibond and Lovibond 1995). Depression is characterized by prolonged periods of low mood, which are accompanied by a series of clinical and physical symptoms and can even lead to suicidal behaviors (Ding et al. 2024; Wang and Huang 2014). According to estimates, 5% of adults worldwide suffer from depression each year, with the highest prevalence among young people (Herrman et al. 2022). Depression has a detrimental impact on cognitive, behavioral, and vegetative functions, leading to a decline in the body's overall functioning (Elkjaer et al. 2022; Gullett et al. 2023; Rock et al. 2014). This, in turn, impairs the individual's capacity to live a normal life and engage in social interactions (Ding et al. 2024; Jin et al. 2024; Zhang 2016). Anxiety and depression often coexist, with a high comorbidity rate; the study has shown that 85% of individuals with depression also experience anxiety symptoms (Gorman 1996). Anxiety is a psychological state characterized by complex emotions such as excessive worry and restlessness, typically arising from an individual's response to an anticipated adverse situation (Tang and Kuang 2009). The result indicated that an increasing number of Chinese college students suffer from anxiety disorders following the COVID‐19 outbreak (W. Z. Li et al. 2022). Anxiety not only profoundly affects the physiological function and psychological state of individuals, but also extensively affects their social well‐being, and then intensifies the overall health pressure and disease burden of society (Nechita et al. 2018). The causes of depression and anxiety are very complex, generally considered the result of interactions among genetic, environmental, social, and other factors (Pehrson and Sanchez 2015; Pitman et al. 2018). Moreover, depression and anxiety are often accompanied by psychological stress, and their three elements are inextricably linked (Juruena et al. 2020; Yang et al. 2015). When college students confront multiple pressures such as academic demands, employment concerns, and interpersonal relationships, these emotions, if not effectively managed, may progressively evolve into depression and anxiety (C. A.‐O. Liu et al. 2019; Pascoe et al. 2020). Therefore, exploring the mechanisms underlying depression, anxiety, and stress is of paramount importance.

Childhood trauma is defined as the negative experiences suffered by individuals before reaching adulthood, including emotional and physical abuse, neglect, and so on, which exceed their ability to cope and cause physical and psychological damage (Cheng 2024; Gürsoy and Mechmet 2023a; Kuzminskaite et al. 2022). The severe psychological trauma caused by childhood trauma may persist into adulthood, leading to mental disorders later in life (Copeland et al. 2018). The shattered assumptions theory posits that individuals, after experiencing a significant trauma, are unable to maintain psychological stability, thereby leading to emotional distress (Janoff‐Bulman 1992). The studies show that childhood trauma ranks as one of the most critical risk factors contributing to the emergence of depressive and anxiety disorders in adulthood (de Bles et al. 2023; Gürsoy and Mechmet 2023; Y. Liu et al. 2024; Peng et al. 2025). Research suggests that childhood trauma is related to abnormalities in brain structure and the HPA axis (the hypothalamic‐pituitary‐adrenal axis), affecting the development of depressive symptoms (Guo et al. 2020). Additionally, childhood trauma has been proven to cause dysregulation of the innate immune system, leading to a greater susceptibility to anxiety disorders (de Koning et al. 2022). Meta‐analyses have shown that childhood trauma is positively correlated with depression and anxiety (Dalechek et al. 2024; Souama et al. 2023; Xiao et al. 2023). Among all traumatic childhood events, victims of sexual, emotional, and physical child abuse are, on average, most likely to experience depressive symptoms, and child sexual abuse and domestic violence are the most likely causes of later anxiety symptoms (De Venter et al. 2013). Some studies suggest that individual psychosocial stress is associated with childhood trauma (Rokita et al. 2021; Shen et al. 2025). Childhood trauma is directly related to depression, anxiety, and stress (Tang et al. 2024), but the underlying mechanisms require further research and discussion.

Alexithymia describes a condition in which patients with psychosomatic illnesses experience impairments or deficiencies at cognitive and emotional levels when recognizing, distinguishing, and communicating their own emotions (Sifneos 2000). A Study has shown that some people who suffered childhood trauma have smaller insular lobe volumes than those who have not experienced trauma (Opel et al. 2019). Since the insula plays an important role in emotional awareness, this may affect an individual's ability to express emotions to some extent (Wei 2023). Neurobiological studies have shown that childhood trauma may result in alterations to the volume of the amygdala (Anda et al. 2006), and changes in amygdala volume have been associated with the progression of alexithymia (Farah et al. 2018). In clinical settings, it is widely acknowledged that most patients diagnosed with alexithymia have a history of emotional abuse or neglect (Honkalampi et al. 2020; Kefeli et al. 2018). The Attention‐Appraisal Model indicates that individuals with higher levels of alexithymia exhibit diminished attention to and evaluation of emotions (Preece et al. 2017), leading to emotional dysregulation and the emergence of emotional distress (Taylor et al. 2024). Moreover, individuals with alexithymia, who experience difficulties in identifying and describing emotional states, are more likely to suffer from anxiety and depression due to their inability to communicate emotions effectively and relieve internal stress (Chen 2016; Dong et al. 2021; Onur et al. 2013). As the level of alexithymia increases, the manifestation of negative emotions like depression and anxiety becomes more evident (Cai et al. 2013; Li et al. 2015). Important research has shown that childhood trauma can indirectly influence individual depression through alexithymia (Ren et al. 2022).

Psychological flexibility refers to an individual's ability to adapt to constantly changing environments by flexibly adjusting their behavior, emotions, and thinking (Landi et al. 2022; Zhang and Dong 2024). It has been found that psychological flexibility is linked to trauma (Boykin et al. 2020). When individuals are traumatized, they draw on a multitude of psychological resources to regulate their responses, which ultimately results in a reduction in psychological flexibility (Tao et al. 2024). Individuals with childhood trauma can influence their well‐being through psychological flexibility (Browne et al. 2022), and lower psychological flexibility is more likely to lead to depression, anxiety, and stress (Landi et al. 2022; Puolakanaho et al. 2023; Shen et al. 2024). According to Acceptance and Commitment Therapy (ACT) theory, cognitive fusion is the core mechanism of psychological inflexibility (Levin et al. 2024), and it is significantly associated with alexithymia (Luminet et al. 2021). Empirical studies have shown that individuals with alexithymia generally have lower levels of psychological flexibility (Edwards and Lowe 2021). This association may stem from their unique patterns of emotion regulation. Individuals with alexithymia often resort to denial and suppression when confronting external emotions, which restricts their ability to flexibly adjust their psychological responses and thereby further exacerbates the impairment of psychological flexibility (Xu and Sun 2018).

Several studies have found a strong link between depression, anxiety, and stress in individuals and childhood trauma, but how specifically childhood trauma affects these mental health problems remains unclear. Therefore, the hypotheses proposed in this study are as follows: (1) Childhood trauma is positively correlated with depression, anxiety, and stress. (2) Alexithymia and psychological flexibility are mediating variables between childhood trauma and depression, anxiety, and stress. (3) Childhood trauma is linked to depression, anxiety, and stress through two successive mediating processes: alexithymia and psychological flexibility. The main aim of this study is to establish a structural model to provide a valuable reference for preventing and intervening depression, anxiety, and stress among college students in the future.

## 2 Methods

### 2.1 Participants

From February to July 2024, questionnaires were distributed to universities in Anhui Province, China, and other universities across the country using online convenience sampling. According to the questionnaire sample size formula (Roco Videla et al. 2021), the sample volume of the cross‐sectional study is estimated to be 500. Some questionnaires were excluded because participants did not answer the security questions correctly. For example, for question 4, “Please select option 5 for this question,” if the participant does not select this option, the questionnaire will be eliminated. Some questionnaires were excluded because the response time was too short and multiple items were selected for the same item. College students enrolled for one to 5 years were all participants in this study, and they all signed an informed consent form. 1002 students total completed the survey (valid questionnaire: 88.32%). The age of the participants ranged from 18 to 27 years, with an average of 21.22 years; 186 were male students (21.02%) and 699 were female students (78.98%); 133 were freshmen (15.03%), 241 were sophomores (27.23%), 206 were juniors (23.28%), and 305 were seniors (34.46%).

**FIGURE 1:**
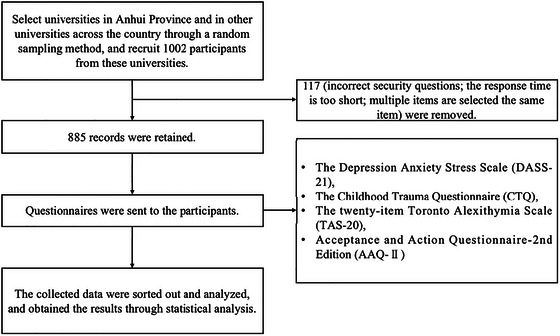
The flowchart of the study. A total of 1002 questionnaires were collected, of which 117 were invalid and the remaining 885 were valid.

All participants were allowed to withdraw from the investigation readily. Informed consent was received from participants for this study by the guidelines set forth by the Ethics Committee of Bengbu Medical University (2024‐411). As demonstrated in Figure 1.

### 2.2 Measurements

#### 2.2.1 General Information Questionnaire

A generalized survey was devised based on the research objectives and interviews with different college students. It was divided into two sections: the first section covered demographic details like gender, grade, age, and educational attainment, while the second section focused on relevant family information, including parents' marital status and perceived connection with their children.

#### 2.2.2 The Depression Anxiety Stress Scale (DASS‐21)

The DASS‐21 (Lovibond and Lovibond 1995) comprises 21 items, divided into three subscales for depression, anxiety, and stress, with each subscale containing seven items. The score for every subscale is a doubled sum of the seven items. For depression, the normal scale is 0–9 points; for anxiety, the normal scale is 0–7 points; for pressure, the normal scale is 0–14 points (Lee 2019). The DASS‐21 demonstrates strong reliability and validity, making it an effective instrument for assessing the levels of negative emotions among Chinese college students (Han et al. 2021). Cronbach's α for the whole scale was 0.930 in the study, 0.837 for the subscale of depression, 0.794 for anxiety, and 0.836 for stress. Higher total scores are indicative of elevated levels of depression, anxiety, and stress.

#### 2.2.3 Childhood Trauma Questionnaire (CTQ)

The CTQ (Bernstein et al. 1994) comprises five key dimensions: emotional abuse, physical abuse, sexual abuse, emotional neglect, and physical neglect. The questionnaire has 28 items in sum (25 clinical items and three validity items), each of which is rated on five grades: 1 = “never true” and 5 = “very often true.” The total score range is 25–125, with scores proportional to the prevalence of childhood trauma. A positive determination is made if any of the following criteria are met: emotional abuse score of 13 or higher, physical abuse score of 10 or higher, sexual abuse score of eight or higher, emotional neglect score of 15 or higher, and physical neglect score of 10 or higher. A positive diagnosis of childhood trauma is indicated if one or more of these criteria are met. The overall Cronbach's α coefficient of the CTQ was 0.887.

#### 2.2.4 The 20‐item Toronto Alexithymia Scale (TAS‐20)

The 20‐item TAS‐20 (Bagby et al. 1994) is a psychometric instrument utilized to assess personality traits in individuals experiencing dysphoria or difficulties in emotional expression. The scale is a self‐assessment questionnaire comprising 20 entries, each of which is rated on five grades, allowing for a total score that ranges from 20 to 100 points. Scores ≤ 51 were categorized as non‐alexithymia disorder (low level of alexithymia disorder), 52–60 as moderate alexithymia disorder, and ≥ 61 as high alexithymia disorder. The TAS‐20 has been demonstrated to be reliable and effective. Cronbach's α coefficient for this questionnaire was 0.786.

#### 2.2.5 Acceptance and Action Questionnaire‐2nd Edition (AAQ‐II)

AAQ‐II (Bond et al. 2011) comprises seven items, each rated on seven grades from 1 (never correct) to 7 (always correct). The total score is obtained by summing the scores of the seven items, resulting in a range from 7 to 49 points. Higher total scores indicate greater experiential avoidance and lower psychological flexibility. The scale has demonstrated satisfactory validity and reliability in previous studies. Cronbach's α coefficient for the whole scale was 0.913.

### 2.3 Procedure

The survey was based on Wenjuanxing online (wjx. cn). The online survey was carried out from February to July 2024. In the initial phase of questionnaire development, the input of professionals was sought, and the direction was then taken and subjected to meticulous review by the instructors. Once the questionnaire had been developed, it was distributed to groups of university students in the form of QR codes and web links, whereupon they completed it carefully and thoroughly. Before responding to the questionnaire, provide an informed consent form to subjects, which outlines the voluntary nature of their participation in the study. Simultaneously, the individuals participating in the study signed a confidentiality agreement. It stipulates that the findings of the measurements cannot be disclosed to the public, that the results of the test are not open to arbitrary interpretation, and that the responses to the questionnaire are utilized exclusively for scientific research purposes. Only after electronically indicating their understanding and consent were students granted access to the survey, which remained inaccessible to non‐consenting individuals.

### 2.4 Data Analysis

The scales selected for this study were well‐established, highly reliable, and technologically advanced in their measurement, mitigating the potential influence of general methodological bias on the results. The data underwent standardization of the transformation process. Subsequently, a parameterization should be conducted on all data. Furthermore, the potential influence of common methodological deviations was addressed through the utilization of a chi‐squared test, complemented by an independent sample *t*‐test for the comparative analysis of two sets of data. Correlation analysis was conducted to examine the link between childhood trauma, alexithymia, psychological flexibility, depression, anxiety, and stress in Chinese college students. Data were statistically analyzed using SPSS 27.0 for Harman's common method bias testing, descriptive statistics, and correlation analyses, and analyzing the mediating effects of psychological flexibility and narrative disorder on the presence of childhood trauma on the depression, anxiety, and stress in Chinese college students using the PROCESS plug‐in for SPSS software. All hypothesis tests were performed using a two‐tailed test, and the difference is significant from a statistical perspective by *p* < 0.05.

## 3 Results

### 3.1 A Holistic Description of Depression, Anxiety, and Stress in College Students

Table 1 summarizes the DASS‐21 scores of 885 college students. Depression scores ranged from 0 to 38 (*M =* 8.00, SD *=* 7.53). Of the total number of participants, 299 exhibited depression scores above the delineated threshold of 9, resulting in a detection rate of 33.79% for depression. Anxiety scores ranged from 0 to 40 (M = 9.08, SD = 7.29). The analysis revealed that 473 individuals exhibited an anxiety score exceeding 7, indicating a recognition rate for anxiety of 53.45%. Stress scores ranged from 0 to 40 (M = 11.25, SD = 8.30). A total of 244 individuals exhibited stress scores that exceeded the delineated threshold of 14, resulting in a stress detection rate of 27.57%. Number of individuals for each trauma subtype: emotional abuse, 104; physical abuse, 95; sexual abuse, 178; emotional neglect, 174; physical neglect, 267. A statistically significant discrepancy was identified between the group with and without childhood trauma on the DASS‐21, TAS‐20, AAQ‐II, and CFQ‐F scale scores (*t* = 6.045, *p* = 0.000; *t* = 7.728, *p* = 0.000; *t* = 4.719, *p* = 0.000; *t* = 3.625, *p* = 0.000).

**TABLE 1:** The detection rate of childhood trauma among college students with different demographic characteristics and Comparison of DASS‐21, TAS‐20, and AAQ‐II scores.

Project	Total students (*n* = 885)	Without childhood trauma (*n* = 420)	With childhood trauma (*n* = 465)	*x* ^2^ */t*	*p*
Gender				33.795	< 0.001
Female	699 (78.98)	314 (74.76)	385 (82.80)		
Male	186 (21.02)	106 (25.24)	80 (17.20)		
Only child				0.300	0.584
Yes	310 (35.03)	151 (35.95)	159 (34.19)		
No	575 (64.97)	269 (64.05)	306 (65.81)		
Home location				3.341	0.068
Landscape	393 (44.41)	200 (47.62)	193 (41.51)		
Cities and towns	492 (55.59)	220 (52.38)	272 (58.49)		
Parental marriage				2.249	0.134
Normal	829 (93.67)	388 (92.38)	441 (94.84)		
Divorced	56 (6.33)	32 (7.62)	24 (5.16)		
Relationship with mother				33.616	< 0.001
Estranged	25 (2.82)	23 (5.48)	2 (0.43)		
Generic	86 (9.72)	72 (17.14)	149 (3.01)		
Close	774 (87.46)	325 (77.38)	449 (96.56)		
Relationship with father				53.126	< 0.001
Estranged	116 (13.11)	82 (19.52)	34 (7.31)		
Generic	162 (18.31)	99 (23.57)	63 (13.55)		
Close	607 (68.59)	239 (59.60)	368 (79.14)		
The number of times I hurt myself in the past 6 months				71.348	< 0.001
Often occur	6 (0.68)	6 (1.43)	0		
Several times (three times and more)	12 (1.36)	9 (2.14)	3 (0.65)		
Twice	22 (2.49)	16 (3.81)	69 (1.29)		
Once	54 (6.10)	46 (10.95)	8 (1.72)		
Without	791 (89.38)	343 (81.67)	448 (96.34)		
M ± SD				*t*	*P*
Depression	8.00 ± 7.53	6.30 ± 6.31	12.33 ± 8.63	7.180	< 0.001
Anxiety	9.08 ± 7.29	7.66 ± 6.25	10.66 ± 8.02	6.148	< 0.001
Stress	11.25 ± 8.30	10.28 ± 7.88	12.33 ± 8.63	3.683	< 0.001
DASS‐21	28.33 ± 21.40	32.87 ± 23.29	24.24 ± 18.64	6.045	< 0.001
TAS‐20	51.05 ± 10.05	53.69 ± 9.06	48.66 ± 10.31	7.728	< 0.001
AAQ‐II	22.40 ± 8.80	23.86 ± 8.95	21.09 ± 8.46	4.719	< 0.001

*Note*: DASS‐21, The depression anxiety stress scale; Depression, depression subscale in the DASS‐21; Anxiety, anxiety subscale in the DASS‐21; Stress, stress subscale in the DASS‐21; TAS‐20, The 20‐item Toronto Alexithymia Scale; AAQ‐II, Acceptance and Action Questionnaire‐2nd Edition; CTQ, Childhood Trauma Questionnaire.

### 3.2 The Correlation Between Childhood Trauma, Depression, Anxiety, Stress, Alexithymia, and Psychological Flexibility in College Students

**FIGURE 2:**
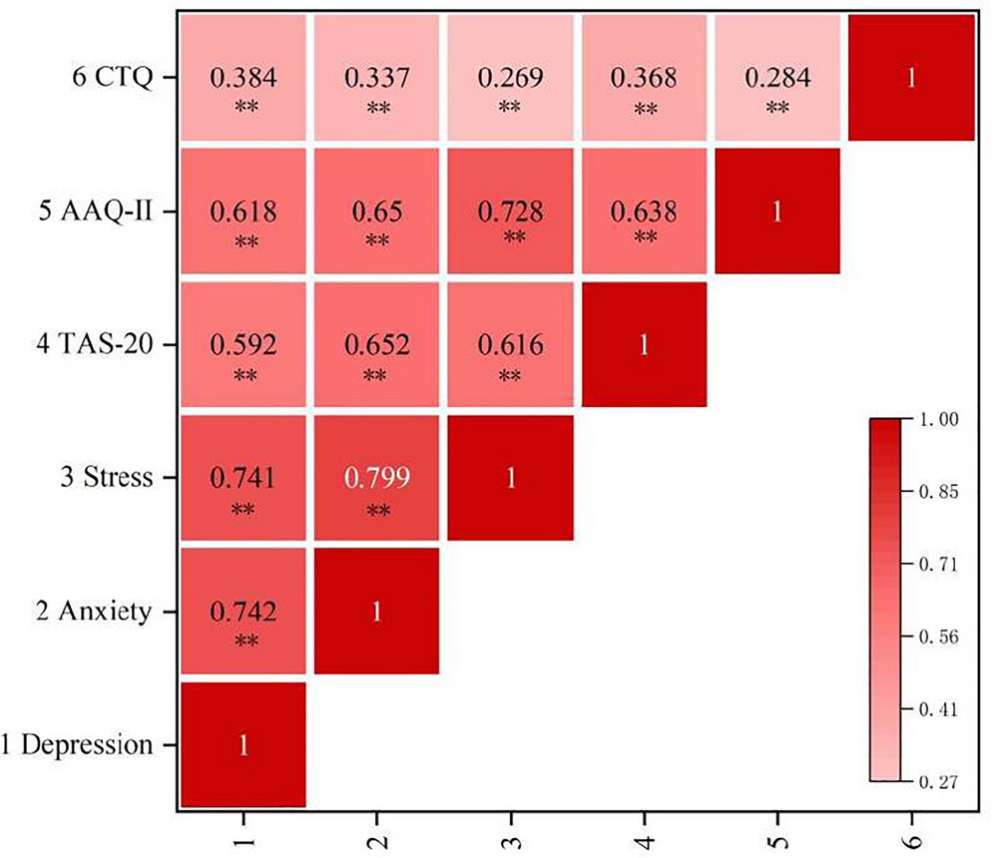
Descriptive statistics and correlation analysis of variables. Depression, depression subscale in the DASS‐21(The Depression Anxiety Stress Scale); Anxiety, anxiety subscale in the DASS‐21 (The Depression Anxiety Stress Scale); Stress, stress subscale in the DASS‐21 (The Depression Anxiety Stress Scale); TAS‐20, The 20‐item Toronto Alexithymia Scale; AAQ‐II, Acceptance and Action Questionnaire‐2nd Edition; CTQ, Childhood Trauma Questionnaire. **p < 0.01.

The results demonstrated that all the variables are positively and significantly correlated (*p* < 0.01). Moreover, depression, anxiety, and stress exhibited a significantly positive correlation with alexithymia and psychological flexibility (*p* < 0.01). As shown in Figure 2.

### 3.3 The Impact of Childhood Trauma on Depression, Anxiety, and Stress: The Chain‐Mediated Effects of Alexithymia and Psychological Flexibility

Following the standardization of the data, the mediated effects of alexithymia and psychological flexibility between childhood trauma and depression, anxiety, and stress were analyzed by Model 6 in the PROCESS plugin. All paths in the model were shown to be significant in the results. In this model, childhood trauma was employed as the independent variable, with depression, anxiety, and stress used as the dependent variables. The mediated variables were alexithymia and psychological flexibility. (As can be observed in Table 2). Depression (*β* = 0.249, *t* = 9.89, 95% CI = 0.124–0.185), anxiety (*β* = 0.170, *t* = 6.92, 95% CI = 0.073–0.132), and stress (*β* = 0.055, *t* = 2.36, 95% CI = 0.006–0.069) were positively associated with childhood trauma in the direct pathway. In the mediated pathway in which alexithymia was used as a mediated variable, childhood trauma was found to be positively associated with alexithymia (*β* = 0.366, *t* = 11.67, 95% CI = 0.252–0.354), and alexithymia was associated with depression (*β* = 0.216, *t* = 6.89, 95% CI = 0.116–0.207), anxiety (*β* = 0.304, *t* = 9.92, 95% CI = 0.177–0.264), and stress (*β* = 0.216, *t* = 7.41, 95% CI = 0.131–0.226) positively. In the mediated pathway with psychological flexibility as the mediated variable, childhood trauma was positively associated with psychological flexibility (*β* = 0.095, *t* = 3.46, 95% CI = 0.030–0.108), and psychological flexibility was positively associated with depression (*β* = 0.433, *t* = 14.08, 95% CI = 0.319–0.422), anxiety (*β* = 0.418, *t* = 13.90, 95% CI = 0.298–0.396), and stress (*β* = 0.585, *t* = 20.40, 95% CI = 0.499–0.605); and in the chain mediation path constituted with alexithymia and psychological flexibility, alexithymia was positively associated with psychological flexibility (*β* = 0.613, *t* = 22.40, 95% CI = 0.490–0.584).

**TABLE 2:** Mediating effects of alexithymia and psychological flexibility on the association between childhood trauma and depression, anxiety, and stress among college students.

Regression equation		Integration fit index			Significance of regression coefficients	
Result variables	Prediction variables	*R*	*R* ^2^	*F*	*β*	*t*
Depression	Childhood trauma	0.465	0.217	244.036	0.465	15.622***
Anxiety	Childhood trauma	0.415	0.172	183.349	0.415	13.541***
Stress	Childhood trauma	0.321	0.103	101.358	0.321	10.068***
Depression	Childhood trauma	0.724	0.524	323.263	0.249	9.892***
	Alexithymia				0.216	6.892***
	Psychological flexibility				0.433	14.081***
Anxiety	Childhood trauma	0.738	0.544	350.136	0.170	6.924***
	Alexithymia				0.304	9.919***
	Psychological flexibility				0.418	13.903***
Stress	Childhood trauma	0.765	0.585	414.506	0.055	2.362*
	Alexithymia				0.216	7.407***
	Psychological flexibility				0.585	20.402***
Alexithymia	Childhood trauma	0.366	0.134	136.119	0.366	11.667***
Psychological flexibility	Childhood trauma	0.654	0.427	329.194	0.095	3.456***
	Alexithymia				0.613	22.403***

*p < 0.05; ***p < 0.001.

**FIGURE 3:**
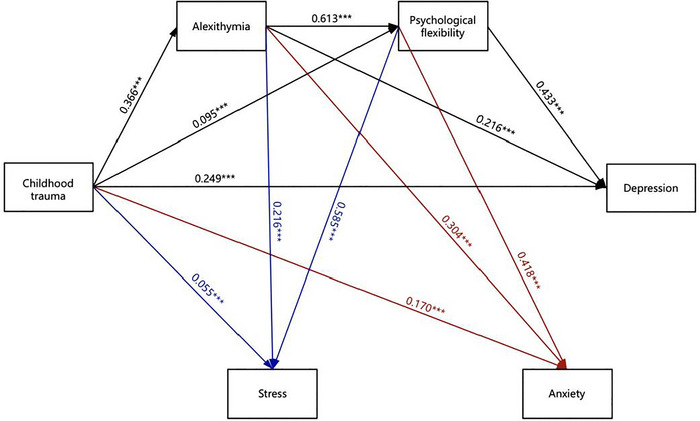
Chain mediating role of narrative disorders and psychological flexibility in the effects of childhood trauma on depression, anxiety, and stress. ***p < 0.001.

As evidenced by the results, relating alexithymia and psychological flexibility fulfilled a significant mediated role between childhood trauma and depression, anxiety, and stress, respectively. Furthermore, the chain mediation effect was remarkable. As demonstrated in Table 3 and Figure 3, indirect effects (46.55%, 58.88%, and 82.74% of the total effects) were achieved by three mediated chains: first, indirect effect one (16.94%, 26.80%, and 24.60%) consisted of childhood trauma → alexithymia → depression, anxiety, and stress; second, indirect effect two (8.80%, 9.50%, and 17.30%) consisted of childhood trauma → psychological flexibility → depression, anxiety, and stress; third, indirect effect three (20.85%, 22.60%, and 40.80%) consisted of childhood trauma → alexithymia → psychological flexibility → depression, anxiety, and stress.

**TABLE 3:** Decomposition of total effect, direct effect, and mediating effect.

Variable		Effect value	Boot standard error	BootLLCI	BootULCI	Relative intermediary effect as a percentage
Depression	Total indirect effect	0.134	0.012	0.111	0.159	46.55%
	Ind1	0.049	0.008	0.034	0.066	16.94%
	Ind2	0.025	0.007	0.011	0.040	8.80%
	Ind3	0.060	0.008	0.046	0.076	20.85%
	Ind1‐Ind2	0.024	0.012	0.002	0.047	
	Ind1‐Ind3	−0.011	0.011	−0.034	0.011	
	Ind2‐Ind3	−0.035	0.011	−0.057	−0.014	
	Direct effect	0.154	0.016	0.124	0.185	53.45%
	Total effect	0.289	0.019	0.252	0.325	
Anxiety	Total indirect effect	0.147	0.013	0.122	0.174	58.88%
	Ind1	0.067	0.009	0.051	0.084	26.8%
	Ind2	0.024	0.007	0.010	0.038	9.5%
	Ind3	0.056	0.007	0.043	0.072	22.6%
	Ind1‐Ind2	0.043	0.011	0.021	0.065	
	Ind1‐Ind3	0.010	0.011	−0.011	0.031	
	Ind2‐Ind3	−0.033	0.011	−0.055	−0.013	
	Direct effect	0.102	0.015	0.073	0.132	41.08%
	Total effect	0.249	0.018	0.213	0.285	
Stress	Total indirect effect	0.182	0.017	0.150	0.215	82.74%
	Ind1	0.054	0.009	0.037	0.074	24.6%
	Ind2	0.038	0.011	0.016	0.059	17.3%
	Ind3	0.090	0.010	0.071	0.111	40.8%
	Ind1‐Ind2	0.016	0.015	−0.012	0.046	
	Ind1‐Ind3	−0.036	0.013	−0.062	−0.011	
	Ind2‐Ind3	−0.052	0.016	−0.085	−0.022	
	Direct effect	0.038	0.016	0.006	0.069	17.26%
	Total effect	0.220	0.022	0.177	0.262	

## 4 Discussion

### 4.1 The Situation and Features of Depression, Anxiety, and Stress Faced by Current College Students

Among the 885 individuals surveyed in this study, the screening rates for depression, anxiety, and stress were 33.79%, 53.45%, and 27.57%, respectively, representing an improvement over those reported in earlier studies (Chen et al. 2023; W. Z. Li et al. 2022). Notably, nearly half of the college students suffered from anxiety. This finding is consistent with previous research conclusions, which collectively highlight the widespread presence of anxiety among Chinese college students in their daily lives and studies (Liao et al. 2024). The high detection rate of anxiety may be related to the outbreak of the new crown epidemic (Hawes et al. 2022), which has brought about tremendous life changes, resulting in the development of bad habits, tensions in interpersonal relationships, confusion regarding career direction, and increased family and academic pressures among college students. Therefore, to promote the healthy and all‐round development of college students, it is essential to understand how to effectively intervene in their symptoms of depression, anxiety, and stress.

### 4.2 Relationship Between Childhood Trauma and Various Variables

In this research, individuals with childhood trauma accounted for 53% of the total sample, indicating that childhood trauma occurs at a significant frequency in the college student population, which was consistent with the examination of some researchers (R. Li et al. 2023). The research data revealed that significant gender differences existed in the childhood trauma experienced by college students, with significantly more female students being exposed to childhood trauma than males. Research indicates that individuals who have experienced childhood trauma significantly differ from those who have not in terms of scores on the three core dimensions of depression, anxiety, and stress. Specifically, individuals with trauma experiences scored significantly higher on the relevant subscales than those without trauma experiences. A correlation analysis study showed a notable positive relationship between childhood trauma and depression, anxiety, and stress among college students, which means that the stronger the trauma an individual suffered in childhood, the more pronounced the symptoms that cause depression, anxiety, and stress later on. Additionally, childhood trauma was also positively correlated with total scores on alexithymia and psychological flexibility.

### 4.3 Analysis of the Mediating Role of Alexithymia and Psychological Flexibility

The results showed that childhood trauma directly influenced depression, anxiety, and stress, accounting for 53.45%, 41.08%, and 17.26% of the overall impact, respectively, and Hypothesis 1 was supported. Depression exhibited the strongest correlation, which may be attributed to the negative perceptions of self and the world that individuals who have experienced childhood trauma tend to develop (Kimble et al. 2018). The cognitive‐behavioral model indicates that negative schemas and cognitions are closely related to the occurrence and maintenance of depressive symptoms (Disner et al. 2011). This may also be because trauma can disrupt the development of an individual's emotion regulation system (Cloitre et al. 2019). According to the emotion regulation dysfunction model, deficits in emotion regulation capacity are characteristic of depression (James 1998). Moreover, individuals with depression tend to more frequently employ negative emotion regulation strategies, and these regulatory deficits perpetuate the cycle of symptoms (Joormann and Stanton 2016). Moreover, this result aligned with the outcomes of previous similar research endeavors (Hoffman et al. 2023).

To investigate how childhood trauma influences depression, anxiety, and stress through alexithymia and mental flexibility, a chain mediation model was constructed. Analysis of the mediating effects showed that alexithymia and psychological flexibility acted as chain mediators between the two. This meant that childhood trauma can indirectly affect depression, anxiety, and stress through alexithymia and psychological flexibility.

Childhood trauma influences depression, anxiety, and stress in a variety of ways; the first is mediated by alexithymia. Previous research have indicated that childhood trauma can cause the amygdala to atrophy (Teicher et al. 2016), and changes in amygdala volume are strongly related to alexithymia (Goerlich‐Dobre et al. 2015). These changes in amygdala volume can lead to dysfunction and cause symptoms of depression and anxiety (P. Hu et al. 2022). Thus, childhood trauma may cause problems in the individual's perception and expression of emotions by affecting brain functioning, which ultimately leads to depression and anxiety through the long‐term production of adverse emotions. The second pathway has psychological flexibility as a mediating variable. In this study, it was found that childhood trauma caused individuals to have reduced psychological flexibility, which contributed to their symptoms of depression and anxiety, which was aligned with earlier findings (Boykin et al. 2020; Landi et al. 2022). The third is a pathway with alexithymia and psychological flexibility as chained mediating variables. Previous research has found that alexithymia can reduce psychological flexibility (Edwards and Lowe 2021). As a result, childhood trauma can cause individuals to have difficulty understanding and expressing emotions effectively. As emotional regulation is impaired, it is difficult for individuals to flexibly adjust their emotions and responses in the face of situational changes. Ultimately, the individual's ability to regulate in the face of negative situations decreases, leading to depression and anxiety.

Previous studies on how childhood trauma affects depression and anxiety among college students are more often, whereas less research has been conducted on childhood trauma and stress. The present study indicated that childhood trauma could also indirectly affect stress through alexithymia and psychological flexibility. This may be because trauma experienced in childhood can cause abnormalities in the HPA axis. Important research found a close correlation between the activity of the HPA axis and the psychological stress experienced by individuals (Godoy et al. 2018; Murphy et al. 2022). Specifically, traumatic experiences in childhood may interfere with the normal regulatory mechanisms of the HPA axis, preventing it from responding appropriately when faced with stress, which in turn increases an individual's stress levels. These three mediating pathways enable hypotheses 2 and 3 to be tested.

By applying the chain‐mediated model, this study reveals how childhood trauma, alexithymia, and psychological flexibility work together to influence depression and anxiety, which can provide new perspectives and methods for mental health interventions for college students. Individuals facing trauma need long‐term attention and follow‐up, including regular assessment using social resources to understand their psychological status and changing needs, as well as timely adjustment of interventions. Eye movement desensitization reprocessing and flicker therapy can be used to improve negative thinking patterns and behavioral habits (S. Hu et al. 2023; Ling et al. 2024), thereby reducing the negative impact of trauma, enhancing psychological flexibility, and alleviating symptoms of depression, anxiety, and stress. Most importantly, it is essential to reduce the occurrence of abusive behaviors at the source and fundamentally alleviate psychological trauma in children. This can be achieved by ensuring that everyone grows up in a safe and supportive environment, which is crucial for the development of a healthy personality and psychological well‐being.

### 4.4 Innovation

This study focuses on the prevalent psychological issues of today. Utilizing theories such as the shattered assumptions theory and the attention‐appraisal model, it explores the relationships among childhood trauma, alexithymia, psychological flexibility, and emotional distress. Methodologically, we first divided the population into two groups—those with and without trauma—for comparative analysis. Subsequently, based on the full sample, we constructed a chain mediation model to comprehensively reveal the systematic associations among the variables.

### 4.5 Limitations

First of all, the participants in this study were randomly sampled from only one school, which limits the representativeness of the findings for all college students; future studies should sample from a broader range of institutions to enhance the generalizability of the results. Second, in this study, there was a large gap between the number of male and female participants, and this unbalanced gender ratio added to the uncertainty of the results. Thirdly, the study employed a cross‐sectional survey and lacked long‐term tracking and observation of the participants, precluding the establishment of clear causal relationships between the variables. Conducting longitudinal surveys in future studies will help to reveal causal associations between variables. Fourth, all the scales used were self‐assessment scales, and the assessed may exaggerate or underestimate their actual situation due to various psychological factors (e.g., self‐esteem, emotional state, etc.), which leads to a deviation of the assessment results from the real state, thus affecting the accuracy of the results.

## 5 Conclusion

Childhood trauma can directly and positively affect the current situation of depression, anxiety, and stress of college students and indirectly affect these psychological states through the path of alexithymia and psychological flexibility. The more traumatic an individual is, the less able they are to express their emotions and the less psychological flexibility they have, resulting in higher levels of depression, anxiety, and stress. Preventing childhood trauma and improving cognition, expression, and mental flexibility can effectively reduce depression, anxiety, and stress. The purpose of this study is to enhance the mental health of college students and build solid theoretical support for their all‐around development and healthy growth.

## Author Contributions

**Ning Wang**: writing – original draft, writing – review and editing, data curation, investigation, methodology, software, formal analysis, visualization, project administration. **Jingyi Chu**: writing – original draft, data curation, investigation. **Guo Chen**: writing – original draft, investigation, visualization. **Xingyue Pan**: investigation. **Ziyue Wang**: investigation. **Wenjuan Wang**: writing – review and editing, funding acquisition, conceptualization, validation, supervision, project administration, resources.

## Ethics Statement

The Ethics Committee of Bengbu Medical University (2024‐411) reviewed and approved the study involving human participants. All data were obtained with the informed consent of the person concerned as well as the legal guardian of the minor.

## Consent

Consent for publication was obtained from the participants.

## Conflicts of Interest

The authors declare no conflicts of interest.

## Peer Review

The peer review history for this article is available at https://publons.com/publon/10.1002/brb3.70825

## Data Availability

The datasets employed and examined during the study can be acquired from the corresponding author upon receipt of a valid request.

## References

[brb370825-bib-0001] Anda, R. F. , V. J. Felitti , J. D. Bremner , et al. 2006. “The Enduring Effects of Abuse and Related Adverse Experiences in Childhood. A Convergence of Evidence From Neurobiology and Epidemiology.” European Archives of Psychiatry and Clinical Neuroscience 256, no. 3: 174–186. 10.1007/s00406-005-0624-4. 16311898 PMC3232061

[brb370825-bib-0002] Bagby, R. M. , J. D. Parker , and G. J. Taylor . 1994. “The Twenty‐Item Toronto Alexithymia Scale–I. Item Selection and Cross‐Validation of the Factor Structure.” Journal of Psychosomatic Research 38, no. 1: 23–32. 10.1016/0022-3999(94)90005-1. 8126686

[brb370825-bib-0003] Bernstein, D. P. , L. Fink , L. Handelsman , et al. 1994. “Initial Reliability and Validity of a New Retrospective Measure of Child Abuse and Neglect.” American Journal of Psychiatry 151, no. 8: 1132–1136. 10.1176/ajp.151.8.1132. 8037246

[brb370825-bib-0004] Bond, F. W. , S. C. Hayes , R. A. Baer , et al. 2011. “Preliminary Psychometric Properties of the Acceptance and Action Questionnaire‐II: A Revised Measure of Psychological Inflexibility and Experiential Avoidance.” Behavior Therapy 42, no. 4: 676–688. 10.1016/j.beth.2011.03.007. 22035996

[brb370825-bib-0005] Boykin, D. M. , J. Anyanwu , K. Calvin , and H. K. Orcutt . 2020. “The Moderating Effect of Psychological Flexibility on Event Centrality in Determining Trauma Outcomes.” Psychol Trauma 12, no. 2: 193–199. 10.1037/tra0000490. 31282720

[brb370825-bib-0006] Brochier, T. , and J. P. Olie . 1993. “Stress and Depression.” Encephale 19, no. 1: 171–178. https://www.ncbi.nlm.nih.gov/pubmed/8281898. 8281898

[brb370825-bib-0007] Browne, A. , O. Stafford , A. Berry , et al. 2022. “Psychological Flexibility Mediates Wellbeing for People With Adverse Childhood Experiences During COVID‐19.” Journal of Clinical Medicine 11, no. 2: 377. 10.3390/jcm11020377. 35054070 PMC8778161

[brb370825-bib-0008] Cai, H. , Z. Li , H. Liu , and X. Chen . 2013. “Alexithymia in Schizophrenia: Association With Executive Function and Emotional Disorder.” Journal of Psychiatry 26, no. 2: 107–110.

[brb370825-bib-0009] Chen, H. 2016. “The Relationship Between Alexithymia and Emotional Symptoms: Mediating Effects of Boredom Proneness.” Chinese Journal of Clinical Psychology 24, no. 4: 648–651. 10.16128/j.cnki.1005-3611.2016.04.016.

[brb370825-bib-0010] Chen, H. G. , H. L. Feng , Y. Y. Liu , et al. 2023. “Anxiety, Depression, Insomnia, and PTSD Among College Students After Optimizing the COVID‐19 Response in China.” Journal of Affective Disorders 337: 50–56. 10.1016/j.jad.2023.05.076. 37245554 PMC10219678

[brb370825-bib-0011] Cheng, L. 2024. “Research Progress on the Effects of Childhood Trauma on Mental Disorders.” Systems Medicine 9, no. 6: 195–198. 10.19368/j.cnki.2096-1782.2024.06.195.

[brb370825-bib-0012] Clark, L. A. , and D. Watson . 1991. “Tripartite Model of Anxiety and Depression: Psychometric Evidence and Taxonomic Implications.” Journal of Abnormal Psychology 100, no. 3: 316–336. 10.1037//0021-843x.100.3.316. 1918611

[brb370825-bib-0013] Cloitre, M. , C. Khan , M. A. Mackintosh , et al. 2019. “Emotion Regulation Mediates the Relationship Between ACES and Physical and Mental Health.” Psychological Trauma: Theory, Research, Practice and Policy 11, no. 1: 82–89. 10.1037/tra0000374. 29745688

[brb370825-bib-0014] Copeland, W. E. , L. Shanahan , J. Hinesley , R. F. Chan , and E. J. Costello . 2018. “Association of Childhood Trauma Exposure With Adult Psychiatric Disorders and Functional Outcomes.” JAMA Network Open 1, no. 7: e184493. 10.1001/jamanetworkopen.2018.4493. 30646356 PMC6324370

[brb370825-bib-0015] Dalechek, D. E. , L. Caes , G. McIntosh , and A. C. Whittaker . 2024. “Anxiety, History of Childhood Adversity, and Experiencing Chronic Pain in Adulthood: A Systematic Literature Review and Meta‐Analysis.” European Journal of Pain 28, no. 6: 867–885. 10.1002/ejp.2232. 38189218

[brb370825-bib-0016] de Bles, N. A.‐O. , L. E. H. Pütz , N. A.‐O. Rius Ottenheim , et al. 2023. “Childhood Trauma and Anger in Adults With and Without Depressive and Anxiety Disorders.” Acta Psychiatrica Scandinavica 148, no. 3: 288–301. 10.1111/acps.13589. 37430486

[brb370825-bib-0017] de Koning, R. M. , E. Kuzminskaite , C. H. Vinkers , E. J. Giltay , and B. Penninx . 2022. “Childhood Trauma and LPS‐Stimulated Inflammation in Adulthood: Results From the Netherlands Study of Depression and Anxiety.” Brain, Behavior, and Immunity 106: 21–29. 10.1016/j.bbi.2022.07.158. 35870669

[brb370825-bib-0018] De Venter, M. , K. Demyttenaere , and R. Bruffaerts . 2013. “[The Relationship Between Adverse Childhood Experiences and Mental Health in Adulthood. A Systematic Literature Review].” Tijdschr Psychiatr 55, no. 4: 259–268. 23595840

[brb370825-bib-0019] Ding, Y. , H. Yuan , X. Chen , D. Zhao , and L. Mao . 2024. “Progress in Modern Medical Treatment of Depression.” Medical Journal of West China 36, no. 4: 614–618.

[brb370825-bib-0020] Disner, S. G. , C. G. Beevers , E. A. Haigh , and A. T. Beck . 2011. “Neural Mechanisms of the Cognitive Model of Depression.” Nature Reviews Neuroscience 12, no. 8: 467–477. 10.1038/nrn3027. 21731066

[brb370825-bib-0021] Dong, J. , J. Zhang , Z. Gao , and S. Yang . 2021. “Research Progress on Alexithymia.” Journal of Psychiatry 34, no. 6: 565–569.

[brb370825-bib-0022] Edwards, D. J. , and R. Lowe . 2021. “Associations Between Mental Health, Interoception, Psychological Flexibility, and Self‐As‐Context, as Predictors for Alexithymia: A Deep Artificial Neural Network Approach.” Frontiers in Psychology 12, no. 1664–1078: 637802.. 10.3389/fpsyg.2021.637802. 33868110 PMC8044902

[brb370825-bib-0023] Elkjaer, E. , M. B. Mikkelsen , J. Michalak , D. S. Mennin , and M. S. O'Toole . 2022. “Motor Alterations in Depression and Anxiety Disorders: A Systematic Review and Meta‐Analysis.” Journal of Affective Disorders 317: 373–387. 10.1016/j.jad.2022.08.060. 36037990

[brb370825-bib-0024] Farah, T. , S. C. Ling , A. Raine , Y. L. Yang , and R. Schug . 2018. “Alexithymia and Reactive Aggression: The Role of the Amygdala.” Psychiatry Research‐Neuroimaging 281: 85–91. 10.1016/j.pscychresns.2018.09.003. 30273792 PMC6226305

[brb370825-bib-0025] Godoy, L. D. , M. T. Rossignoli , P. Delfino‐Pereira , N. Garcia‐Cairasco , and E. H. de Lima Umeoka . 2018. “A Comprehensive Overview on Stress Neurobiology: Basic Concepts and Clinical Implications.” Frontiers in Behavioral Neuroscience 12: 127. 10.3389/fnbeh.2018.00127. 30034327 PMC6043787

[brb370825-bib-0026] Goerlich‐Dobre, K. S. , M. Votinov , U. Habel , J. Pripfl , and C. Lamm . 2015. “Neuroanatomical Profiles of Alexithymia Dimensions and Subtypes.” Human Brain Mapping 36, no. 10: 3805–3818. 10.1002/hbm.22879. 26094609 PMC6869665

[brb370825-bib-0027] Gorman, J. M. 1996. “Comorbid Depression and Anxiety Spectrum Disorders.” Depression and Anxiety 4, no. 4: 160–168. 10.1002/(SICI)1520-6394(1996)4:4<160::AID-DA2>3.0.CO;2-J. 9166648

[brb370825-bib-0028] Gullett, N. , Z. Zajkowska , A. Walsh , R. Harper , and V. Mondelli . 2023. “Heart Rate Variability (HRV) as a Way to Understand Associations Between the Autonomic Nervous System (ANS) and Affective States: A Critical Review of the Literature.” International Journal of Psychophysiology 192: 35–42. 10.1016/j.ijpsycho.2023.08.001. 37543289

[brb370825-bib-0029] Guo, W. , J. Liu , and L. Li . 2020. “Major Depressive Disorder With Childhood Trauma:Clinical Characteristics, Biological Mechanism, and Therapeutic Implications.” Journal of Central South University(Medical Science) 45, no. 4: 462–468. 10.11817/j.issn.1672-7347.2020.190699 32879073

[brb370825-bib-0030] Gürsoy, M. Y. , and F. C. Mechmet . 2023. “Correlations Between Childhood Trauma and Depression, Anxiety, and Stress Levels in Nurses.” Archives of Psychiatric Nursing 45: 164–168. 10.1016/j.apnu.2023.06.018. 37544694

[brb370825-bib-0031] Han, T. , W. Ma , H. Gong , et al. 2021. “Investigation and Analysis of Negative Emotion Among University Students During Home Quarantine of COVID‐19.” Journal of Xi'an Jiaotong University (Medical Sciences) 42, no. 1: 132–136.

[brb370825-bib-0032] Hawes, M. T. , A. K. Szenczy , D. N. Klein , G. Hajcak , and B. D. Nelson . 2022. “Increases in Depression and Anxiety Symptoms in Adolescents and Young Adults During the COVID‐19 Pandemic.” Psychological Medicine 52, no. 14: 3222–3230. 10.1017/S0033291720005358. 33436120 PMC7844180

[brb370825-bib-0033] Herrman, H. , V. Patel , C. Kieling , et al. 2022. “Time for united Action on Depression: A Lancet‐World Psychiatric Association Commission.” Lancet 399, no. 10328: 957–1022. 10.1016/S0140-6736(21)02141-3. 35180424

[brb370825-bib-0034] Hoffman, S. N. , M. B. Stein , and C. T. Taylor . 2023. “Childhood Trauma Predicts Positive Expressive Suppression during Social Affiliation in Adults with Anxiety and/or Depression: Implications for Social Functioning.” Behavior Therapy 54, no. 2: 375–385. 10.1016/j.beth.2022.10.003. 36858766 PMC10911195

[brb370825-bib-0035] Honkalampi, K. , N. Flink , S. M. Lehto , et al. 2020. “Adverse Childhood Experiences and Alexithymia in Patients With Major Depressive Disorder.” Nordic Journal of Psychiatry 74, no. 1: 45–50. 10.1080/08039488.2019.1667430. 31808358

[brb370825-bib-0036] Hu, P. , Y. Lu , B. X. Pan , and W. H. Zhang . 2022. “New Insights Into the Pivotal Role of the Amygdala in Inflammation‐Related Depression and Anxiety Disorder.” International Journal of Molecular Sciences 23, no. 19: Article 11076. 10.3390/ijms231911076. 36232376 PMC9570160

[brb370825-bib-0037] Hu, S. , W. Ou , Z. Wang , and J. Peng . 2023. “Eye Movement Desensitization and Reprocessing versus Sertraline in the Treatment of Depressed Adolescents With Childhood Trauma.” Chinese General Practice 26, no. 6: 692–698.

[brb370825-bib-0038] James, J. G. 1998. “The Emerging Field of Emotion Regulation: An Integrative Review.” Review of General Psychology 2, no. 3: 271–299. 10.1037/1089-2680.2.3.271.

[brb370825-bib-0039] Janoff‐Bulman, R. 1992. Shattered Assumptions: Toward a New Psychology of Trauma. Free Press.

[brb370825-bib-0040] Jin, X. , W. Dong , Y. Yan , F. Zhou , T. Gao , and K. Chang . 2024. “Research Progress on Sini San and Its Single Active Ingredient in Anti‐Depressant.” Chinese Archives of Traditional Chinese Medicine 42: 1–17.

[brb370825-bib-0041] Joormann, J. , and C. H. Stanton . 2016. “Examining Emotion Regulation in Depression: A Review and Future Directions.” Regour Research and Therapy 86: 35–49. 10.1016/j.brat.2016.07.007. 27492851

[brb370825-bib-0042] Juruena, M. F. , F. Eror , A. J. Cleare , and A. H. Young . 2020. “The Role of Early Life Stress in HPA Axis and Anxiety.” Advances in Experimental Medicine and Biology 1191: 141–153. 10.1007/978-981-32-9705-0_9. 32002927

[brb370825-bib-0043] Kefeli, M. C. , R. G. Turow , A. Yıldırım , and M. Boysan . 2018. “Childhood Maltreatment Is Associated With Attachment Insecurities, Dissociation and Alexithymia in Bipolar Disorder.” Psychiatry Research 260: 391–399. 10.1016/j.psychres.2017.12.026. 29253803

[brb370825-bib-0044] Kimble, M. , A. Sripad , R. Fowler , S. Sobolewski , and K. Fleming . 2018. “Negative World Views After Trauma: Neurophysiological Evidence for Negative Expectancies.” Psychological Trauma 10, no. 5: 576–584. 10.1037/tra0000324. 30188159 PMC6544388

[brb370825-bib-0045] Kuzminskaite, E. , C. H. Vinkers , Y. Milaneschi , E. J. Giltay , and B. Penninx . 2022. “Childhood Trauma and Its Impact on Depressive and Anxiety Symptomatology in Adulthood: A 6‐Year Longitudinal Study.” Journal of Affective Disorders 312: 322–330. 10.1016/j.jad.2022.06.057. 35760192

[brb370825-bib-0046] Landi, G. , K. I. Pakenham , E. Crocetti , E. Tossani , and S. Grandi . 2022. “The Trajectories of Anxiety and Depression During the COVID‐19 Pandemic and the Protective Role of Psychological Flexibility: A Four‐Wave Longitudinal Study.” Journal of Affective Disorders 307: 69–78. 10.1016/j.jad.2022.03.067. 35378147 PMC8972980

[brb370825-bib-0047] Lee, D. 2019. “The Convergent, Discriminant, and Nomological Validity of the Depression Anxiety Stress Scales‐21 (DASS‐21).” Journal of Affective Disorders 259: 136–142. 10.1016/j.jad.2019.06.036. 31445339

[brb370825-bib-0048] Levin, M. E. , J. Krafft , and M. P. Twohig . 2024. “An Overview of Research on Acceptance and Commitment Therapy.” Psychiatric Clinics of North America 47, no. 2: 419–431. 10.1016/j.psc.2024.02.007. 38724128

[brb370825-bib-0049] Li, R. , L. Ji , and S. Liu . 2023. “The Relationship Between Childhood Trauma and Anxiety and Depression in College Students: The Moderating Effect of Social Support.” Psychological Monthly 18, no. 21: 72–75. 10.19738/j.cnki.psy.2023.21.016.

[brb370825-bib-0050] Li, S. , B. Zhang , Y. Guo , and J. Zhang . 2015. “The Association Between Alexithymia as Assessed by the 20‐Item Toronto Alexithymia Scale and Depression: A Meta‐Analysis.” Psychiatry Research 227, no. 1: 1–9. 25769520 10.1016/j.psychres.2015.02.006

[brb370825-bib-0051] Li, W. Z. , Z. Y. Zhao , D. J. Chen , Y. Peng , and Z. X. Lu . 2022. “Prevalence and Associated Factors of Depression and Anxiety Symptoms Among College Students: A Systematic Review and Meta‐Analysis.” Journal of Child Psychology and Psychiatry 63, no. 11: 1222–1230. 10.1111/jcpp.13606. 35297041

[brb370825-bib-0052] Liao, H. , J. Du , and R. Xiao . 2024. “Relationship Among Anxiety, Perceived Stress and Forbearance in College Students.” Chinese Mental Health Journal 38, no. 3: 277–278.

[brb370825-bib-0053] Ling, H. , H. Liu , W. Zhou , Y. Yan , and X. Huang . 2024. “Intervention of Flash Technique on Anxiety of College Students With Childhood Trauma.” Chinese Journal of Clinical Psychology 32, no. 1: 222–226. 10.16128/j.cnki.1005-3611.2024.01.041.

[brb370825-bib-0054] Liu, C. A.‐O. , C. A.‐O. Stevens , S. H. M. Wong , M. A.‐O. Yasui , and J. A.‐O. Chen . 2019. “The Prevalence and Predictors of Mental Health Diagnoses and Suicide Among U.S. college Students: Implications for Addressing Disparities in Service Use.” Depression and Anxiety 36, no. 1: 8–17. 10.1002/da.22830. 30188598 PMC6628691

[brb370825-bib-0055] Liu, Y. , Q. Shen , L. Duan , L. Xu , Y. Xiao , and T. Zhang . 2024. “The Relationship Between Childhood Psychological Abuse and Depression in College Students: A Moderated Mediation Model.” BMC Psychiatry 24, no. 1: 410. 10.1186/s12888-024-05809-w. 38816793 PMC11141024

[brb370825-bib-0056] Lovibond, P. F. , and S. H. Lovibond . 1995. “The Structure of Negative Emotional States: Comparison of the Depression Anxiety Stress Scales (DASS) With the Beck Depression and Anxiety Inventories.” Behaviour Research and Therapy 33, no. 3: 335–343. 10.1016/0005-7967(94)00075-u. 7726811

[brb370825-bib-0057] Luminet, O. A.‐O. , K. A.‐O. Nielson , and N. A.‐O. Ridout . 2021. “Having no Words for Feelings: Alexithymia as a Fundamental Personality Dimension at the Interface of Cognition and Emotion.” Cognition & Emotion 35, no. 3: 435–448. 10.1080/02699931.2021.1916442. 33900884

[brb370825-bib-0058] Murphy, F. , A. Nasa , D. Cullinane , et al. 2022. “Childhood Trauma, the HPA Axis and Psychiatric Illnesses: A Targeted Literature Synthesis.” Front Psychiatry 13: 748372. 10.3389/fpsyt.2022.748372. 35599780 PMC9120425

[brb370825-bib-0059] Nechita, D. , F. Nechita , and R. Motorga . 2018. “A Review of the Influence the Anxiety Exerts on Human Life.” Romanian Journal of Morphology and Embryology 59, no. 4: 1045–1051. https://www.ncbi.nlm.nih.gov/pubmed/30845283. 30845283

[brb370825-bib-0060] Onur, E. , T. Alkin , M. J. Sheridan , and T. N. Wise . 2013. “Alexithymia and Emotional Intelligence in Patients With Panic Disorder, Generalized Anxiety Disorder and Major Depressive Disorder.” Psychiatric Quarterly 84, no. 3: 303–311. 10.1007/s11126-012-9246-y. 23076764

[brb370825-bib-0061] Opel, N. , R. Redlich , K. Dohm , et al. 2019. “Mediation of the Influence of Childhood Maltreatment on Depression Relapse by Cortical Structure: A 2‐Year Longitudinal Observational Study.” Lancet Psychiatry 6, no. 4: 318–326. 10.1016/S2215-0366(19)30044-6. 30904126

[brb370825-bib-0062] Pascoe, M. C. , S. E. Hetrick , and A. G. Parker . 2020. “The Impact of Stress on Students in Secondary School and Higher Education.” International Journal of Adolescence and Youth 25, no. 1: 104–112. 10.1080/02673843.2019.1596823.

[brb370825-bib-0063] Pehrson, A. L. , and C. Sanchez . 2015. “Altered Gamma‐Aminobutyric Acid Neurotransmission in Major Depressive Disorder: A Critical Review of the Supporting Evidence and the Influence of Serotonergic Antidepressants.” Drug Design, Development and Therapy 9: 603–624. 10.2147/DDDT.S62912. 25653499 PMC4307650

[brb370825-bib-0064] Peng, J. , Y. Liu , X. Wang , Z. Yi , L. Xu , and F. Zhang . 2025. “Physical and Emotional Abuse With Internet Addiction and Anxiety as a Mediator and Physical Activity as a Moderator.” Scientific Reports 15, no. 1: 2305. 10.1038/s41598-025-85943-x. 39824886 PMC11742654

[brb370825-bib-0065] Pitman, A. , S. Suleman , N. Hyde , and A. Hodgkiss . 2018. “Depression and Anxiety in Patients With Cancer.” BMJ‐British Medical Journal 361: k1415. 10.1136/bmj.k1415. 29695476

[brb370825-bib-0066] Preece, D. , R. Becerra , A. Allan , K. Robinson , and J. Dandy . 2017. “Establishing the Theoretical Components of Alexithymia via Factor Analysis: Introduction and Validation of the Attention‐Appraisal Model of Alexithymia.” Personality and Individual Differences 119: 341–352. 10.1016/j.paid.2017.08.003.

[brb370825-bib-0067] Puolakanaho, A. A.‐O. , J. S. Muotka , R. Lappalainen , P. Lappalainen , R. Hirvonen , and N. A.‐O. Kiuru . 2023. “Adolescents' Stress and Depressive Symptoms and Their Associations With Psychological Flexibility Before Educational Transition.” Journal of Adolescence 95, no. 5: 990–1004. 10.1002/jad.12169. 36960576

[brb370825-bib-0068] Ren, L. , Y. Yang , S. Zhang , and W. Fu . 2022. “Relationship Between Childhood Abuse and Depression in Junior Middle School Students: A Chain Intermediary Between Alexithymia and Self Compassion.” China Journal of Health Psychology 30, no. 11: 1734–1740. 10.13342/j.cnki.cjhp.2022.11.024.

[brb370825-bib-0069] Rock, P. L. , J. P. Roiser , W. J. Riedel , and A. D. Blackwell . 2014. “Cognitive Impairment in Depression: A Systematic Review and Meta‐Analysis.” Psychological Medicine 44, no. 10: 2029–2040. 10.1017/S0033291713002535. 24168753

[brb370825-bib-0070] Roco Videla, Á. , M. Hernández Orellana , and O. Silva González . 2021. “What is the Appropriate Sample Size to Validate A Questionnaire?” Nutricion Hospitalaria 38, no. 4: 877–878. 10.20960/nh.03633. 34041917

[brb370825-bib-0071] Rokita, K. I. , M. R. Dauvermann , D. Mothersill , et al. 2021. “Current Psychosocial Stress, Childhood Trauma and Cognition in Patients With Schizophrenia and Healthy Participants.” Schizophrenia Research 237: 115–121. 10.1016/j.schres.2021.08.030. 34521038

[brb370825-bib-0072] Shen, Q. , H. Wang , M. Liu , et al. 2025. “The Impact of Childhood Emotional Maltreatment on Adolescent Insomnia: A Chained Mediation Model.” BMC Psychology 13, no. 1: 506. 10.1186/s40359-025-02803-z. 40369687 PMC12079810

[brb370825-bib-0073] Shen, Q. , S. Wang , Y. Liu , Z. Wang , C. Bai , and T. Zhang . 2024. “The Chain Mediating Effect of Psychological Inflexibility and Stress Between Physical Exercise and Adolescent Insomnia.” Scientific Reports 14, no. 1: 24348. 10.1038/s41598-024-75919-8. 39420219 PMC11486977

[brb370825-bib-0074] Sifneos, P. E. 2000. “Alexithymia, Clinical Issues, Politics and Crime.” Psychotherapy and Psychosomatics 69, no. 3: 113–116. 10.1159/000012377. 10877675

[brb370825-bib-0075] Souama, C. , F. Lamers , Y. Milaneschi , et al. 2023. “Depression, Cardiometabolic Disease, and Their Co‐Occurrence After Childhood Maltreatment: An Individual Participant Data Meta‐Analysis Including Over 200,000 Participants.” BMC Medicine 21, no. 1: Article 93. 10.1186/s12916-023-02769-y. 36907864 PMC10010035

[brb370825-bib-0076] Tang, H. , and C. Kuang . 2009. “Summary of Anxiety Theories.” Chinese Journal of Clinical Psychology 17, no. 2: 176–177+199.

[brb370825-bib-0077] Tang, Q. , X. Zou , J. Gui , et al. 2024. “Effects of Childhood Trauma on the Symptom‐Level Relation Between Depression, Anxiety, Stress, and Problematic Smartphone Use: A Network Analysis.” Journal of Affective Disorders 358: 1–11. 10.1016/j.jad.2024.05.018. 38705521

[brb370825-bib-0078] Tao, J. , L. Liu , X. Li , and Y. Wang . 2024. “A Study on the Relationship Between Parenting Style, Psychological Flexibility, and Aggressive Behavior Among Middle School Students.” Psychological Monthly 19, no. 5: 40–43. 10.19738/j.cnki.psy.2024.05.013.

[brb370825-bib-0079] Taylor, G. J. , P. Porcelli , and R. M. Bagby . 2024. “Alexithymia: A Defense of the Original Conceptualization of the Construct and a Critique of the Attention‐Appraisal Model.” Clin Neuropsychiatry 21, no. 5: 329–357. 10.36131/cnfioritieditore20240501. 39540074 PMC11555664

[brb370825-bib-0080] Teicher, M. H. , J. A. Samson , C. M. Anderson , and K. Ohashi . 2016. “The Effects of Childhood Maltreatment on Brain Structure, Function and Connectivity.” Nature Reviews Neuroscience 17, no. 10: 652–666. 10.1038/nrn.2016.111. 27640984

[brb370825-bib-0081] Wang, R. , and S. Huang . 2014. “The Research Progress of Pathogenesis in Depression.” Journal of Medical Postgraduates 27, no. 12: 1332–1336. 10.16571/j.cnki.1008-8199.2014.12.023.

[brb370825-bib-0082] Wei, R. 2023. “Research Progress in Neuroimaging of Alexithymia.” Journal of Neyroscience and Mental Health 23, no. 11: 827–831.

[brb370825-bib-0083] Xiao, Z. , M. Murat Baldwin , S. C. Wong , I. Obsuth , F. Meinck , and A. L. Murray . 2023. “The Impact of Childhood Psychological Maltreatment on Mental Health Outcomes in Adulthood: A Systematic Review and Meta‐Analysis.” Trauma, Violence & Abuse 24, no. 5: 3049–3064. 10.1177/15248380221122816. PMC10594835 36123796

[brb370825-bib-0084] Xu, F. , and J. Sun . 2018. “A Review of Alexithymia in Elderly Patients With Chronic Diseases.” Chinese Journal of Nursing 53, no. 01: 105–109.

[brb370825-bib-0085] Yang, L. , Y. Zhao , Y. Wang , et al. 2015. “The Effects of Psychological Stress on Depression.” Current Neuropharmacology 13, no. 4: 494–504. 10.2174/1570159x1304150831150507. 26412069 PMC4790405

[brb370825-bib-0086] Zeng, Y. , C. H. Hu , Y. Z. Li , et al. 2024. “Association Between Pretreatment Emotional Distress and Immune Checkpoint Inhibitor Response in Non‐Small‐Cell Lung Cancer.” Nature Medicine 30, no. 6: 1680–1688. 10.1038/s41591-024-02929-4. PMC11186781 38740994

[brb370825-bib-0087] Zhang, Q. 2016. *Behavioral and Electrophysiological Study of Impaired Interpersonal Function of Depression* [Master's thesis, Anhui Medical University].

[brb370825-bib-0088] Zhang, Y. , and L. Dong . 2024. “Childhood Trauma and Psychological Flexibility in Adolescent Patients With Depressive Disorder Relationship to Non‐Suicidal Self‐Injurious Behavior.” Today Nurse 31, no. 5: 127–131. 10.19791/j.cnki.1006-6411.2024.13.032.

